# Induced effects of electrical muscle stimulation and visual stimulation on visual sensory reweighting dynamics during standing on a balance board

**DOI:** 10.1371/journal.pone.0285831

**Published:** 2023-05-22

**Authors:** Masato Shindo, Takashi Isezaki, Yukio Koike, Ryosuke Aoki

**Affiliations:** NTT Human Informatics Laboratories, Nippon Telegraph and Telephone Corporation, Yokosuka, Kanagawa, Japan; Tokai University, JAPAN

## Abstract

Providing instruction cues on body motions using stimulations has the potential to induce sensory reweighting dynamics. However, there are currently very few quantitative investigations on the difference in the induced effects on the sensory reweighting dynamics between stimulation methods. We therefore investigated the difference in the induced effects of electrical muscle stimulation (EMS) and visual sensory augmentation (visual SA) on sensory reweighting dynamics during standing on a balance board. Twenty healthy participants controlled their posture to maintain the board horizontally in the balance-board task, which included a pre-test without stimulation, a stimulation test, and a post-test without stimulation. The EMS group (n = 10) received EMS to the tibialis anterior or soleus muscle based on the board tilt. The visual SA group (n = 10) received visual stimuli via a front monitor based on the board tilt. We measured the height of the board marker and calculated the board sway. Before and after the balance-board task, the participants performed static standing with their eyes open and closed. We measured postural sway and calculated the visual reweighting. The visual reweighting showed a strong negative correlation with the balance board sway ratio between the pre- and stimulation tests in the EMS group and a strong positive correlation with that in the visual SA group. Moreover, for those who reduced the balance board sway in the stimulation test, the visual reweighting was significantly different between the stimulation methods, demonstrating that the induced effect on sensory reweighting dynamics is quantitatively different depending on which method is used. Our findings suggest that there is an appropriate stimulation method to change to the targeted sensory weights. Future investigations on the relationship between sensory reweighting dynamics and stimulation methods could contribute to the proposal and implementation of new training methods for learning to control the target weights.

## Introduction

Human postural control is performed by the sensory integration of the central nervous system to reduce body sway. In this process, the visual, vestibular, and proprioceptive systems detect information on body motion from the external world, and the relative weights of each sensory system change depending on environmental conditions, known as sensory reweighting [1–3]. Maladaptation of sensory reweighting to sudden changes in environmental conditions could lead to postural instability, resulting in falls [4] and motion-sickness [5, 6]. Numerous studies have demonstrated that the change in sensory weights, known as sensory reweighting dynamics, will differ depending on the nature of the subjects. Even if subjects are categorized within the same group (e.g., healthy, elderly, or stroke patients), the reweighting dynamics within each group may differ considerably [7–10]. Therefore, it is important to consider approaches to improve postural stability according to the individual characteristics in the reweighting dynamics.

Providing additional information as instruction cues for body motions to complement and/or replace native sensory inputs could enhance the sensory reweighting dynamics and help improve postural stability [11]. Recent studies have demonstrated a reduction in postural sway using stimulations, such as by sensory augmentation (SA), in various modalities (e.g., vibrotactile [11], visual [12], auditory [13, 14], and electrotactile [15]). In addition, a few reports have discussed sensory reweighting dynamics that result in improved postural stability [11, 16]. Balance training in which unstable surface standing and weight shifting etc. were performed with vibrotactile SA around the trunk showed a substantial increase in reliance on vestibular inputs for healthy older adults [11]. Inspired by these findings, further investigations on induced positive effects on sensory reweighting dynamics by other stimulation methods could be expected in the future. However, the observed effects of the stimulation method on the reweighting dynamics may be context-specific (training task, intervention periods, target population, etc.) [17], which makes it difficult to compare the effect among multiple methods. In this study, we therefore investigate the quantitative differences in the induced effects on sensory reweighting dynamics between stimulation methods instead. This is the first attempt to elucidate the relationship between stimulation methods and induced effects on reweighting dynamics, and our hope is that it contributes to the proposal and implementation of a new training method that enables users to select an appropriate stimulation method to control the target sensory weights. The new training method will ideally provide an optimal intervention based on individual characteristics in the reweighting dynamics and be applicable to a wide range of areas including fall prevention, adaptation to physical disorders, sports training, and motion-sickness prevention.

Our study focuses on two stimulation methods: visual SA and electrical muscle stimulation (EMS). Visual SA, which is the primary method utilized in SA studies, visually provides cues to reduce postural sway by means of a monitor or similar device. One factor contributing to improved postural balance and sensory reweighting with the use of visual SA seems to be the addition of a feedback loop of stimulus perception through vision, recognition, and postural correction based on the postural cues of the SA [17]. As for EMS, it not only provides motor commands but also has unique features that contract muscles and induce involuntary movements to help users learn to move limbs kinematically by controlling their movements directly [18]. Recently, systems that apply EMS to ankle dorsiflexion or plantarflexion muscles during standing have demonstrated postural improvement in healthy subjects [19] and in patients with spinal cord injuries [20]. In contrast to the SA systems mentioned above, EMS-based systems not only provide instruction cues about appropriate motions but also induce involuntary movements to reduce postural sway. Therefore, we expect that these systems will be helpful for those who cannot appropriately control ankle movements even when using SA systems.

The purpose of this study is to investigate the difference in the induced effects of EMS and visual SA on the sensory reweighting dynamics during standing on a balance board rotating in the anteroposterior direction. We utilized a single-leg stance on the balance board as a motor task to simulate a challenging condition that requires rapid sensory reweighting [21]. To maintain the balance board tilt horizontally under the stimulation conditions, EMS was applied to the ankle dorsiflexion or plantarflexion muscles and visual SA was implemented to present appropriate ankle motions on the front monitor. We utilized the stimulus presentation on ankle motion to promote the use of the ankle joint because the ankle joint makes a bigger contribution to improved balance ability with training on a balance board than the hip joint [22]. We analyzed the difference in visual reweighting dynamics and postural sway change between EMS and visual SA and then analyzed the difference in the induced effects on the reweighting dynamics between each stimulation method using visual reweighting dynamics before and after the balance board task. The main contribution of this study is the clarification of the difference in the induced effects of EMS and visual SA on the sensory reweighting dynamics owing to the different roles played by each.

## Methods

### Participants

Twenty healthy males without neurological or musculoskeletal disorders participated in this experiment from 19 to 25 January 2022. They were randomly assigned to the EMS group (n = 10, age = 34.2 ± 9.3 years old, height = 170.2 ± 7.2 cm, weight = 65.9 ± 12.6 kg) or the visual SA group (n = 10, age = 32.8 ± 7.6 years old, height = 174.1 ± 6.9 cm, weight = 68.0 ± 8.1 kg). All participants were informed of the purpose of the experiment, the experimental protocol, and potential risks before signing an informed consent form. This study was reviewed and approved by the ethics committee of the Cybernetics Laboratory of NTT Human Informatics Laboratories (approval number: 2021–02, date of approval: 7th January 2022). The authors did not have access to information that could identify individual participants during or after data collection.

### Task and apparatus

**Fig 1:**
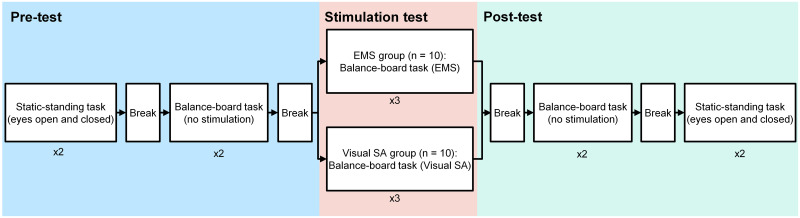
Experimental flow. Participants performed a pre-test, stimulation test, and post-test. The pre- and post-tests consisted of the static-standing task with eyes open and also with eyes closed, and the balance-board task without stimulation. Each task was repeated twice. The stimulation test included the balance-board task with EMS or Visual SA. This task was repeated three times.

To evaluate the effects of each stimulation method, pre- and post-tests were performed before and after the stimulation test (Fig 1). The pre- and post-tests consisted of static-standing and balance-board tasks. We estimated the visual weights in the static-standing tasks and calculated postural sway in the balance-board tasks. In the stimulation test, the balance-board task was performed with one stimulation method (EMS or Visual SA) (see C. Stimulation Methods), and the postural sway was calculated. The postural sway in the balance-board task in the post-test was not used because we focused on the immediate effects of stimulus presentation on postural control in the stimulation test.

#### Static-standing task

The participants stood upright on a force plate (BP400600, AMTI) in bare feet, with their eyes open and then closed. They were instructed to align the heels of their left and right feet, open their toes at 45°, and cross their arms in front of the chest while standing. The force plates computed the center of pressure (COP) position at 1 kHz, which is representative of postural sway. To measure pelvic sway, cameras hooked up to 11 Vicon motion capture systems (Vicon, Vicon Motion Systems) tracked the tri-axial positions of four infrared reflective markers, which were placed on the right anterior pelvis, left anterior pelvis, right posterior pelvis, and left posterior pelvis, at 100 Hz.

#### Balance-board task

The participants stood upright on their bare feet in a right single-leg stance on a 350-mm-diameter balance board (Dyjoc board, SAKAI Medical) between parallel bars (TB-534–02, Takada bed). The balance board was set to rotate only in the anteroposterior direction by attaching three hemispherical parts at the bottom. The center of the board was adjusted to approximately 40% of the foot length from the heel line based on a previous study that investigated the COP in a static-standing position on a stable surface [23]. This adjustment results in zero ankle joint moments when the board is horizontal, i.e., it requires ankle joint muscle coordination to keep the board horizontal. The participants were instructed to keep the balance board as horizontal as possible, with their arms crossed in front of the chest and looking at a black dot on the front monitor. Before the task, they practiced the single-leg stance for 1 min. Those who could not maintain this stance (two in the EMS group and one in the visual SA group) performed a double-leg stance instead.

**Fig 2:**
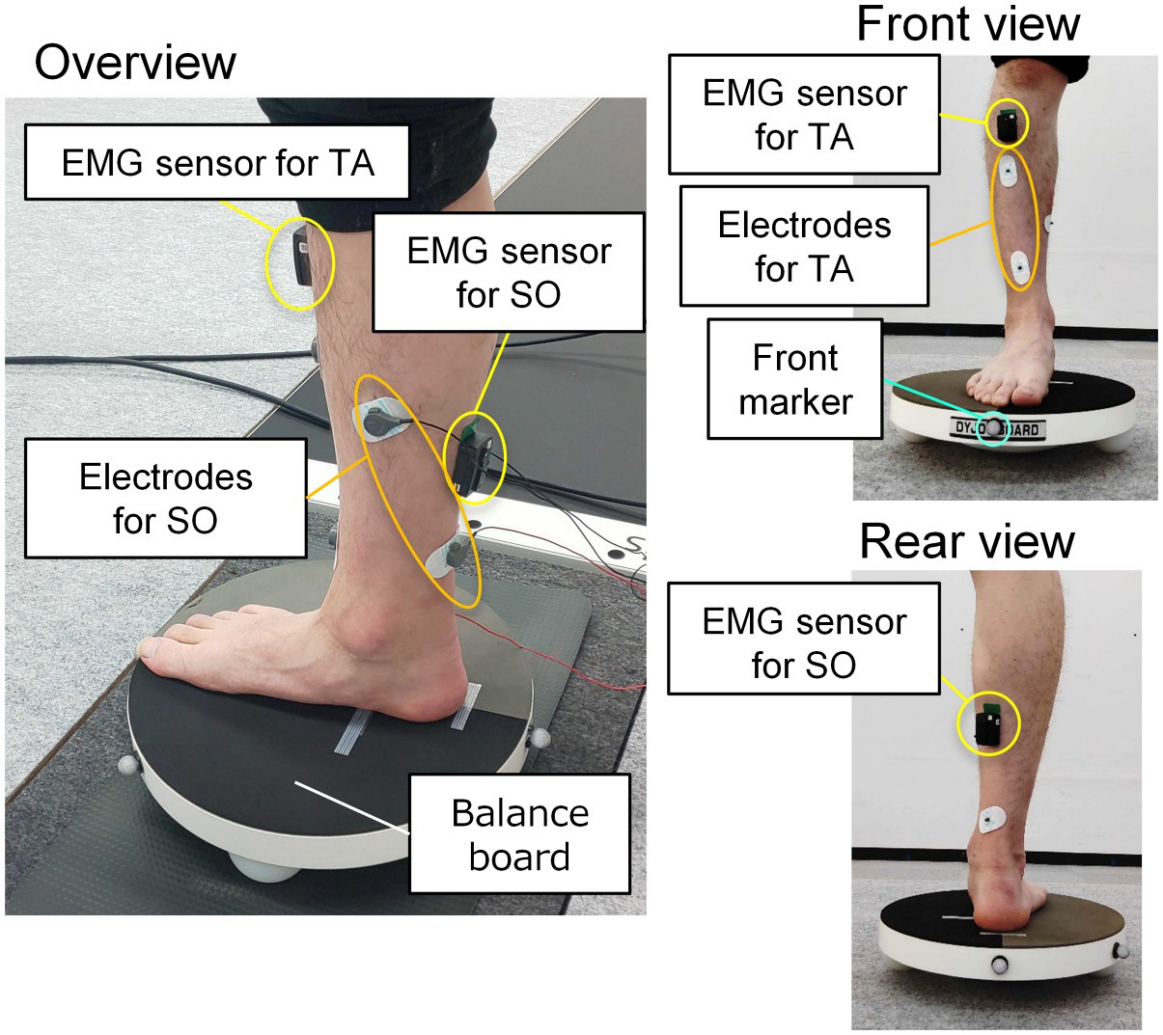
Positions of EMG sensors, EMS electrode (only EMS group), and balance-board markers. The EMG sensors and electrode (only EMS group) were attached to the tibialis anterior muscle (TA) and soleus muscle (SO). Each EMS electrode was sized 30 mm × 15 mm, and only the front marker of the balance board was used.

To measure the balance-board sway during the balance-board task, motion-capture markers were attached to the front, middle, and back ends of the balance board, and the tri-axial positions of the markers were tracked at a sampling frequency of 100 Hz. We only used the height of the front end of the marker (Fig 2). To measure pelvic sway, the tri-axial positions of the four markers around the pelvis were recorded at 100 Hz.

To assess muscle activity in the ankle, electromyography (EMG) sensors (Delsys, Trigno Wireless System) were attached to the tibialis anterior (TA) and soleus (SO) muscles, as shown in Fig 2. We focused on the SO muscle among the triceps surae muscles (gastrocnemius and soleus muscles) because the contribution of its muscle activity is considered crucial, especially in high-difficulty balance tasks such as the balance-board task [24]. The data were collected at a sampling frequency of 1 kHz, and the EMG and motion capture systems were synchronized.

### Stimulation methods

#### EMS group

**Fig 3:**
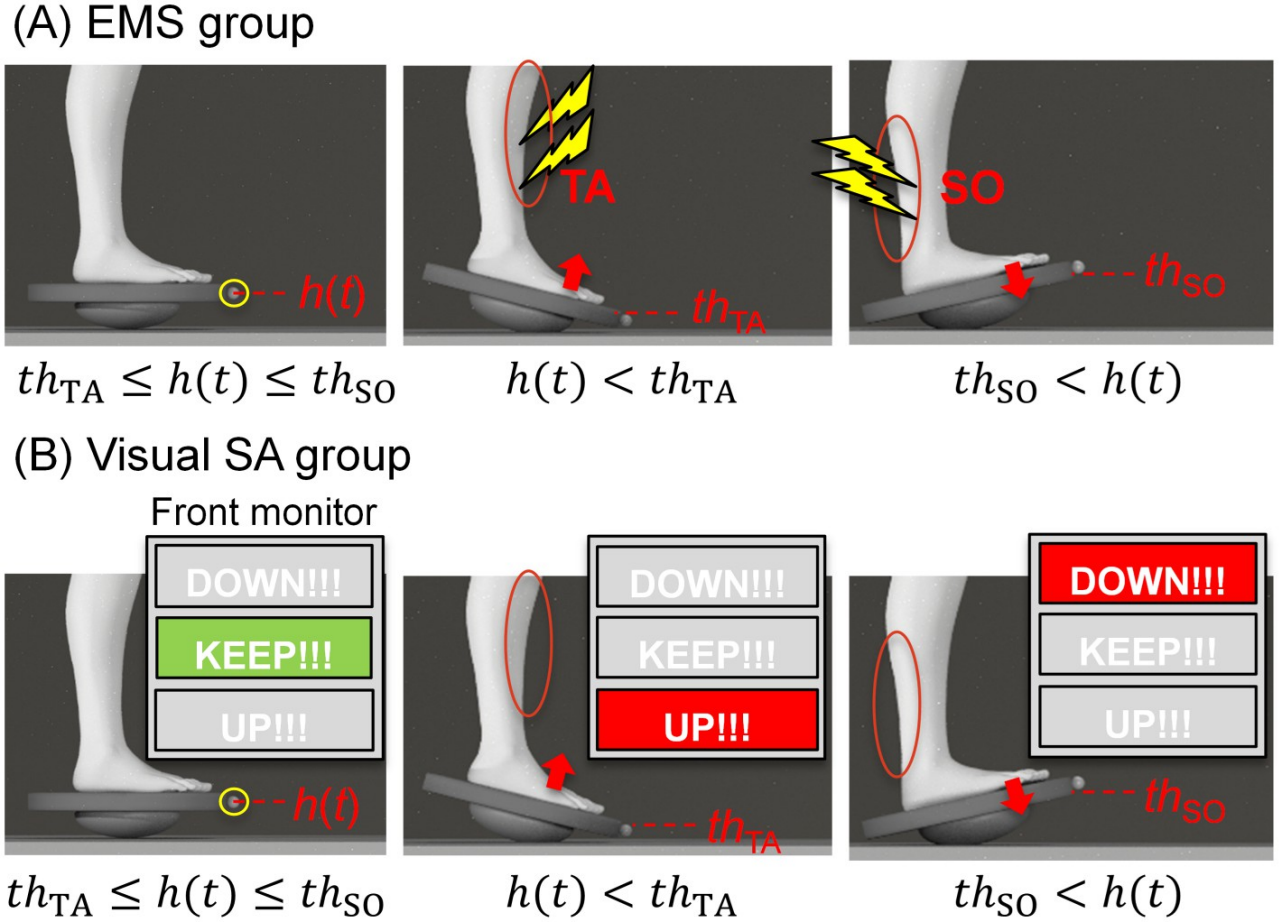
Schematic diagram of stimulation methods for (A) EMS and (B) visual SA groups. The EMS group received EMS to either the TA or SO to control the horizontal balance-board tilt based on the height of the marker of the balance board (*h*). The visual SA group received a visual SA stimulation related to ankle motion to horizontally tilt the balance board based on the height of the marker of the balance board.

**Fig 4:**
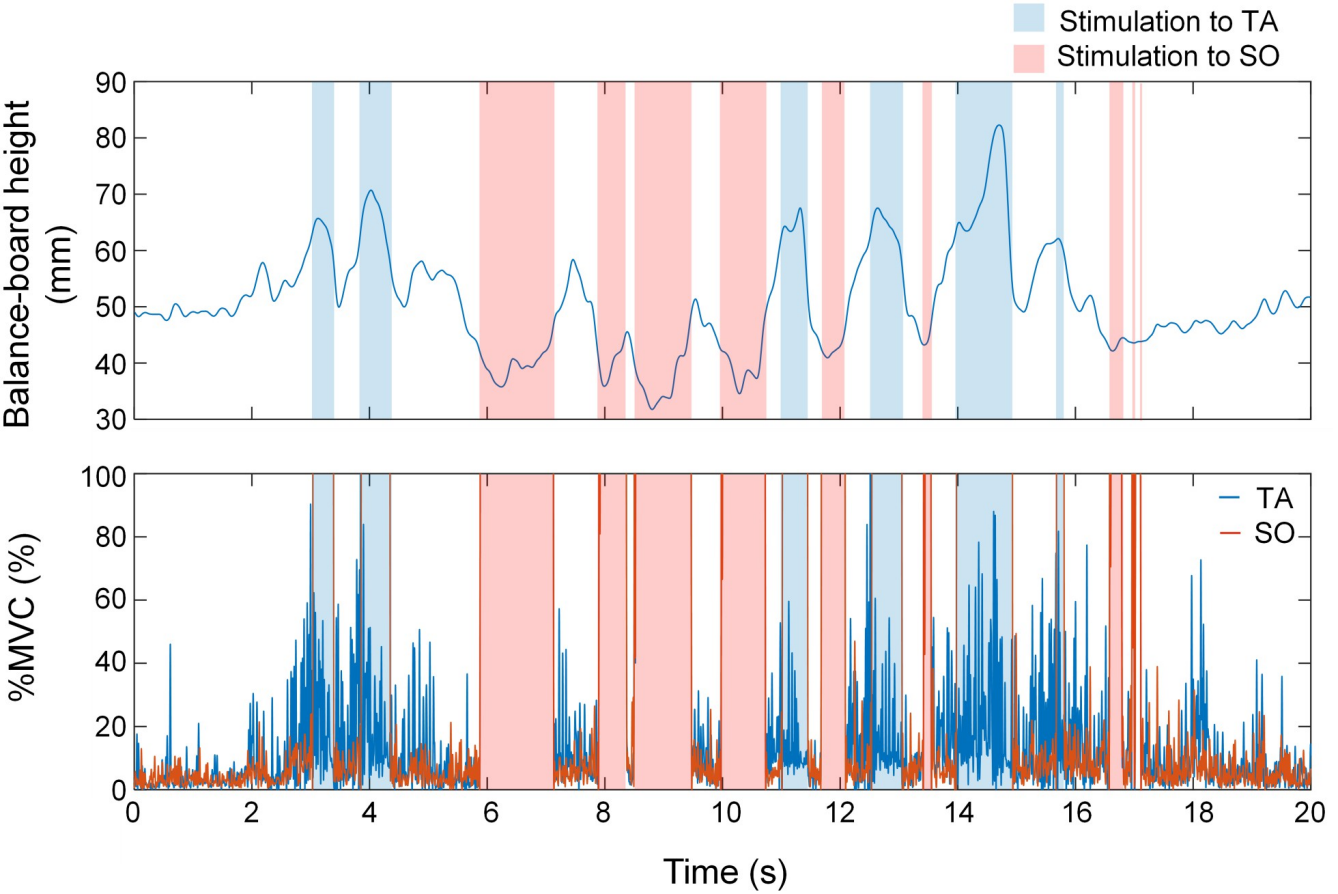
An example of time-series changes of balance-board height (upper), muscle activities (%MVC) (lower), and stimulus timing of EMS in stimulation test for EMS group. The blue area indicates presenting EMS to TA when the board height was below *th*_*TA*_, and the red area indicates presenting EMS to SO when the board height was above *th*_*SO*_.

The participants were equipped with two pairs of disposable electrodes (F-150M, Nihon Kohden), one for the TA and one for the SO of their right leg (Fig 2). Each electrode pair was connected to a channel of the function generator (SEN-8203, Nihon Kohden) through an isolator (SS104-J, Nihon Kohden). The external output of each channel of the function generator was connected to a microcontroller (Arduino Uno, Arduino) to send signals to the function generator and provide stimulation based on the motion of the balance board. The motion-capture system transmitted real-time triggers of the motion data to a microcontroller. We used the height of the balance-board marker (*h*) as motion data. The participants received EMS to the TA or SO based on the two thresholds of the height of the balance board (*th*_*TA*_, *th*_*SO*_), as shown in Fig 3(a). When the height was below *th*_*TA*_, the trigger was sent to the channel connected to the TA, and EMS was applied to the TA. When the height was above *th*_*SO*_, the trigger was sent to the channel connected to the SO, and EMS was applied to the SO. When the height was between *th*_*TA*_ and *th*_*SO*_, the EMS was turned off. *th*_*TA*_ and *th*_*SO*_ were calculated in advance from the results of the balance-board task in the pre-test, as follows:

$$\begin{array}{c} th_{TA}=h_{mean}-h_{std},\;th_{SO}=h_{mean}+h_{std}, \end{array}$$ (1)

where *h*_*mean*_ is the average height of the marker recorded for 40 s (20 s each × 2 trials; the first and final 5 s were excluded), and *h*_*std*_ is the standard deviation of the heights of the markers. The participants were instructed to keep the balance board within the area where EMS was not applied. Fig 4 (upper) shows an example of the time-series changes of the balance-board height (*h*) and stimulus timing of EMS in the stimulation test for the EMS group.

Prior to the experiment, the stimulus intensity was calibrated for each participant. The participants increased the output of the EMS until dorsiflexion or plantarflexion of the ankle was confirmed within a range of no pain from the EMS. Based on the JIS standard (electric therapy apparatus for home use, JIS C 9335–2-209:2018) and the design guidelines [25], we set the pulse width to 200 μs, the frequency to 200 Hz, and the maximum current to 20 mA.

#### Visual SA group

Participants received visual stimuli based on the two thresholds of the height of the balance board (*th*_*TA*_ and *th*_*SO*_) from the monitor positioned in front of them, as shown in Fig 3(b). The display on the monitor replaced the black dot with the instruction of the motor output of the ankle motion. The trigger for motion data transmitted from the motion-capture system was interpreted by a microcontroller and then sent to the PC to present the visual stimulus. When the height of the marker was below *th*_*TA*_, the “UP!!!” button turned red at the bottom of the monitor. When the height was above *th*_*SO*_, the “DOWN!!!” button turned red on the top of the monitor. When the height was between *th*_*TA*_ and *th*_*SO*_, the “KEEP!!!” button turned light green in the middle of the monitor. *th*_*TA*_ and *th*_*SO*_ were calculated with (1) using the results of the balance-board task in the pre-test, as in the EMS group. The participants were instructed to keep the balance board within the current area when the “KEEP!!!” button was turned on.

### Procedure

Fig 1 shows the experimental flow. As stated earlier, this experiment consisted of a pre-test, stimulation test, and post-test. Prior to the experiment, participants performed maximum voluntary muscle contractions (MVCs) of plantarflexion and dorsiflexion of the ankle. To obtain the MVC value in the TA, they sat in a chair with the hip, knee, and ankle at 90° and raised their right toe with maximum force using the TA, while the experimenter opposed the movement by placing a hand over the foot. To obtain the MVC value in the SO, they similarly raised their right heel with maximum force using the SO. Each MVC task was performed twice every 3 s.

In the pre-test, the participants alternately performed static-standing tasks with their eyes open and closed twice. After a 2-min break, which was spent sitting, they performed a balance-board task without stimulation twice. Between each trial, they took a 1-min break. After the pre-test, they took a 2-min break and then performed a stimulation test (balance-board task with stimulation) three times. Between each trial, they took a 1-min break. After that, they took a 2-min break and then performed a post-test, which reversed the order of the static-standing and balance-board tasks from the pre-test.

In the balance-board task, if participants could not hold their posture for 30 s, they grabbed parallel bars to prevent falling, and the trial was repeated after the break.

### Analysis

All raw data were analyzed using MATLAB 2022a. All data from the first and final 5 s were eliminated to avoid anticipation effects associated with the beginning and end of the trial [26]. The COP and motion-capture data were low-pass filtered using a 4th-order zero-lag Butterworth filter with a cut-off frequency of 10 Hz.

#### Visual reweighting

In the quantification of the visual weight (*W*_*vis*_), we first calculated the root mean square (RMS) of the COP trajectories as the COP sway, as

$$\begin{array}{c} COP_{sway}=\sqrt{\frac{1}{n}\sum_{i=1}^{n}\left\{{\left\{COP_{x}\left(i\right)-\bar{COP_{x}}\right\}}^{2}+{\left\{COP_{y}\left(i\right)-\bar{COP_{y}}\right\}}^{2}\right\}}, \end{array}$$ (2)

where *COP*_*x*_(*i*) and *COP*_*y*_(*i*) represent the positions of the COP in the frontal and sagittal planes in the *i*-th sample, respectively, and $\bar{COP_{x}}$ and $\bar{COP_{y}}$ represent the mean positions of the COP for a 20-second-analysis interval. The COP could not be measured for three individuals in the visual SA group because of a problem with the measurement of the force plate. Instead of the COP RMS, the pelvis sway was calculated for the determination of the visual weight, as

$$\begin{array}{c} Pelvis_{sway}=\sqrt{\;\frac{1}{n}\;\sum_{i=1}^{n}\;\left\{{\left\{COM_{x}\left(i\right)\;-\;\bar{COM_{x}}\right\}}^{2}\;+\;{\left\{COM_{y}\left(i\right)\;-\;\bar{COM_{y}}\right\}}^{2}\;+\;{\left\{COM_{z}\left(i\right)\;-\;\bar{COM_{z}}\right\}}^{2}\right\}}, \end{array}$$ (3)

where *COM*_*x*_(*i*), *COM*_*y*_(*i*), and *COM*_*z*_(*i*) represent the amplitudes of the center of the four pelvis markers in the *i*-th sample. $\bar{COM_{x}}$, $\bar{COM_{y}}$, and $\bar{COM_{z}}$ express the mean amplitude of the COM for a 20-second-analysis interval. The COP and pelvis sways were calculated using the data obtained from the static-standing tasks for open and closed eyes in the pre- and post-tests, respectively. Subsequently, we calculated the visual weight (*W*_*vis*_) by calculating the sway ratio under open-eyes and closed-eyes conditions (Romberg rate [27]) as follows:

$$W_{vis}=\frac{COP_{sway}^{EC}}{COP_{sway}^{EO}},$$ (4)

where ${COP}_{sway}^{EC}$ is the mean value of the COP sway in two trials of the closed-eyes condition and ${COP}_{sway}^{EO}$ is that of the open-eyes condition. For three individuals who obtained pelvic sway instead of COP sway, similar to (3), *W*_*vis*_ was calculated using ${Pelvis}_{sway}^{EC}$ and ${Pelvis}_{sway}^{EO}$. Finally, the visual reweighting (*RW*_*vis*_) was quantified by dividing the visual weight in the post-test ($W_{vis}^{post}$) by that in the pre-test ($W_{vis}^{pre}$).

$$\begin{array}{c} RW_{vis}=\frac{W_{vis}^{post}}{W_{vis}^{pre}} \end{array}$$ (5)

#### Postural sway

To assess postural sway, we calculated the RMS of the balance-board sway (*BB*_*sway*_) using the samples of the height of the balance-board marker (*h* = [*h*_1_, …, *h*_*n*_]) for 20 s (upper side of Fig 4) as follows:

$$\begin{array}{c} BB_{sway}=\sqrt{\frac{1}{n}\sum_{i=1}^{n}{\left\{h_{i}-\bar{h}\right\}}^{2}}, \end{array}$$ (6)

where *h*_*i*_ is the *i*-th sample of *h*, and $\bar{h}$ is the mean height of *h*. *BB*_*sway*_ of each trial in each stimulation test and pre-test was calculated to assess sway changes associated with immediate motor alterations due to stimulation. The balance-board sway ratio (*BBSR*) was quantified by dividing the balance-board sway from the stimulation test ($BB_{sway}^{stim}$) by that in the pre-test ($BB_{sway}^{pre}$).

$$\begin{array}{c} BBSR=\frac{BB_{sway}^{stim}}{BB_{sway}^{pre}} \end{array}$$ (7)

#### EMG

To verify the motor alterations associated with each stimulation, we analyzed the EMG of the TA and SO. We applied a 4th-order zero-lag band-pass Butterworth filter (20–450 Hz) and then further rectified and applied a 4th-order zero-lag low-pass Butterworth filter at 50 Hz [28]. For the MVC tasks, we set the maximum EMG value as the MVC value. Subsequently, data were normalized to the MVC value (%MVC). Fig 4 (lower side) shows an example of the time-series changes of %MVC and the stimulus timing of EMS in the stimulation test for the EMS group. To assess changes in %MVC immediately before the start and after the end of each stimulus in the stimulation test, the median values of %MVC were calculated from 1 ms to 100 ms immediately before the start of the stimulus and from 100 ms to 200 ms immediately after the end of the stimulus. We did not extract data from 1 ms to 100 ms immediately after the end of the stimulus because the noise generated by EMS affected the %MVC up to 100 ms immediately after the stimulus. If the median %MVC was greater than 100%, the data was considered to still have the EMS noise and was excluded. The duration of each stimulus was calculated from the raw data on the height of the marker on the balance board. Because the EMG data could not be measured for three individuals (one in the EMS group and two in the visual SA group) owing to a problem with the measurement of the EMG sensors, their %MVC results were excluded.

### Statistical analysis

To verify the induced effects on sensory reweighting in the postural stabilization task, the relationship between visual reweighting and the balance-board sway ratio for the EMS and visual SA groups was evaluated. We calculated the Pearson correlation coefficient to explore the association between visual reweighting (*RW*_*vis*_) and the balance-board sway ratio (*BBSR*) in the pre-and stimulation tests for the EMS and visual SA groups. In the linear regression model, the observed plot indicating that the corresponding residual was larger than the 95% confidence interval was regarded as an outlier, and it was excluded from later evaluation. We divided those whose balance-board sway ratio was less than one (improved participants) from those whose balance-board sway ratio was more than one (deteriorated participants). For comparison of results, we utilized the Wilcoxon non-parametric test, which is applicable with or without data normality. For improved participants, the differences in visual reweighting (*RW*_*vis*_) among the groups were compared using the Wilcoxon rank-sum test. Then, the change in the visual weight (*W*_*vis*_) between pre- and post-tests was compared using the Wilcoxon signed-rank test for improved participants in the EMS and visual SA groups. For a detailed analysis to clarify the influence of each stimulation on visual reweighting, the %MVC values immediately before the start of stimulus and those immediately after the end of stimulus were compared using the Wilcoxon signed-rank test, and the duration of each stimulation in the stimulation test between the improved and deteriorated participants in the EMS and Visual SA groups was compared using the Wilcoxon rank-sum test. The statistical significance for rejecting the null hypothesis was set to *p* < 0.05.

## Results

### Tendencies in visual reweighting dynamics

**Fig 5:**
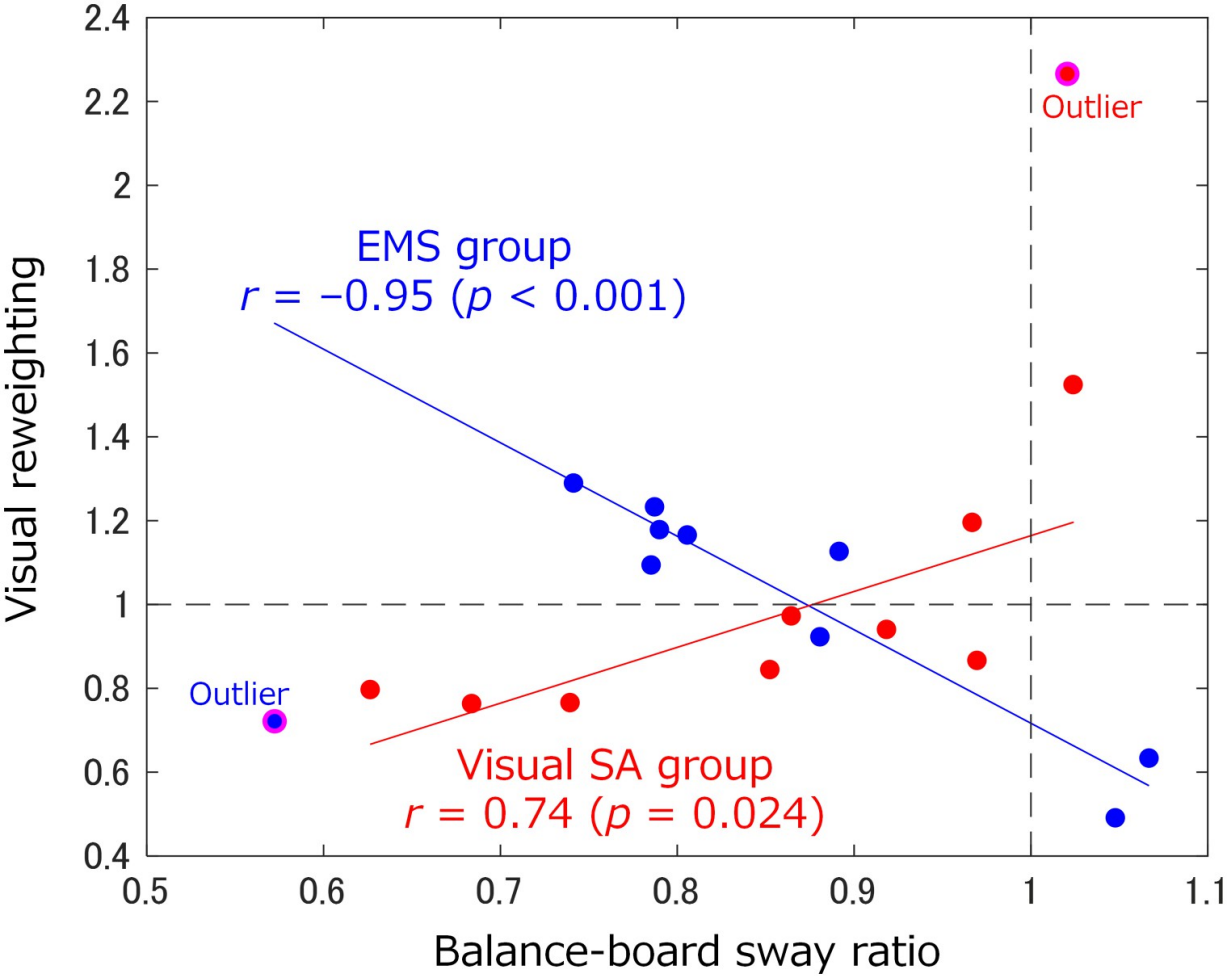
Relationship between visual reweighting (*RW*_*vis*_) and the balance-board sway ratio (*BBSR*) for EMS and visual SA groups. Blue and red lines indicate the linear regression in the EMS and visual SA groups, respectively. Pink circle indicates an outlier identified by the linear regression model. Balance-board sway ratio < 1: postural sway improved; board sway ratio > 1: postural sway deteriorated. Visual reweighting > 1: enhanced visual dependence; reweighting < 1: weakening visual dependence.

Fig 5 shows the relationship between visual reweighting (*RW*_*vis*_) and the balance-board sway ratio (*BBSR*) for the EMS and visual SA groups. The horizontal axis represents the balance-board sway ratio between the pre- and stimulation tests in the balance-board tasks. A ratio of less than one indicates that the balance-board sway in the stimulation test was reduced compared to that in the pre-test. The vertical axis indicates visual reweighting between the pre- and post-tests in the static-standing tasks. An area less than one indicates visual down-weighting in the post-test, and an area greater than one indicates visual up-weighting in the post-test. The null hypothesis, indicating no linear correlation between visual reweighting and the balance-board sway ratio, was rejected with *p* < 0.001 in the EMS group and *p* = 0.024 in the visual SA group, indicating a linear correlation between them in both groups. The result of the Pearson correlation coefficient (*r*) showed a strong negative correlation (*r* = −0.95) in the EMS group and a strong positive correlation (*r* = 0.74) in the visual SA group. The samples regarded as outliers (one sample each in the EMS and visual SA groups) were excluded from later evaluation. From these results, we confirmed the different tendencies in the visual reweighting dynamics according to each stimulation method.

**Fig 6:**
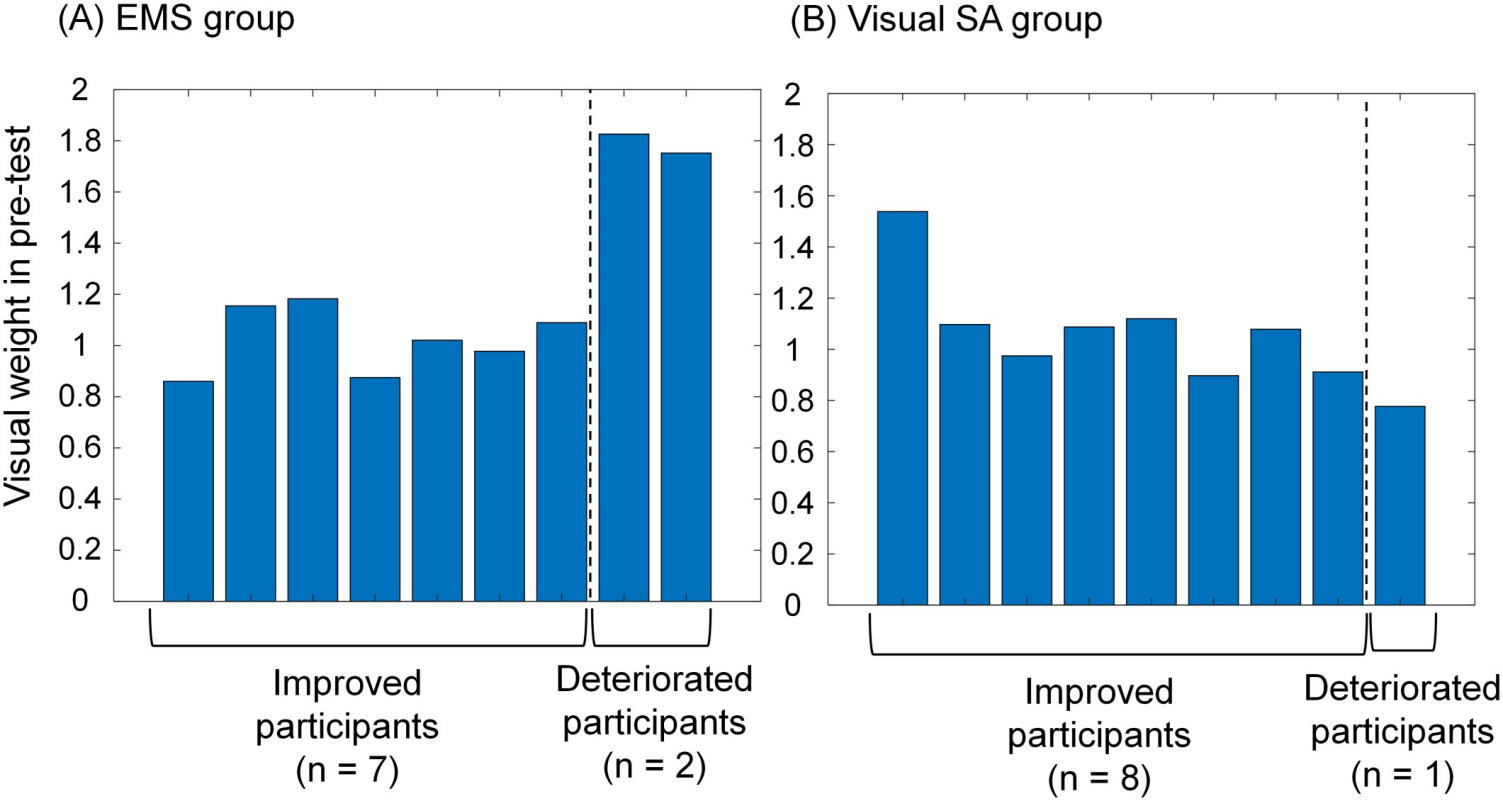
Visual weight of improved and deteriorated participants in the pre-test ($W_{vis}^{pre}$) of the static-standing tasks in (A) EMS group and (B) visual SA group.

Fig 6 shows the visual weight of the improved and deteriorated participants in the pre-test ($W_{vis}^{pre}$) of the static-standing tasks in the (A) EMS group and (B) visual SA group. The deteriorated participants had the highest and second highest visual weights of all participants in the EMS group, whereas the deteriorated participants had the lowest visual weights of all participants in the visual SA group. The postural improvement achieved by stimulation varies among individuals and is strongly associated with the original sensory weights before stimulation [29]. Thus, we speculate that the sensory reweighting dynamics differed between the improved and deteriorated participants.

**Fig 7:**
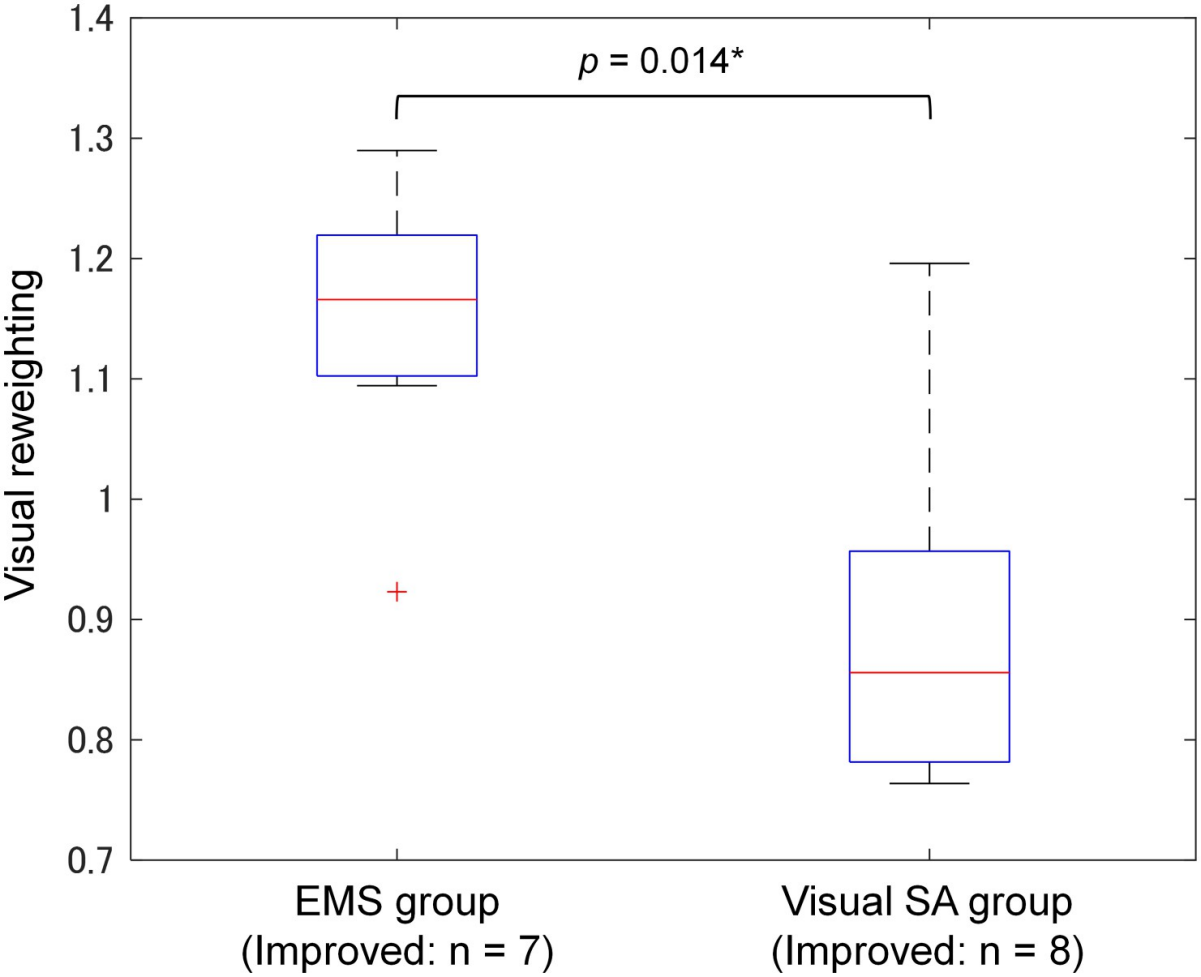
Box plots of visual reweighting (*RW*_*vis*_) between pre-test and post-test. The horizontal line within the box indicates the median, and the bottom and top edges of the box indicate the 25th and 75th percentiles, respectively. Visual reweighting was quantified by dividing the visual weight in the post-test by that in the pre-test. Postural improvement was defined as a reduction in balance-board sway in the stimulation test compared to the pre-test (balance-board sway ratio less than one).

We assessed visual reweighting for the improved participants. Fig 7 shows the comparison of the visual reweighting (*RW*_*vis*_) for the improved participants between the EMS group (n = 7) and visual SA group (n = 8). Using the binomial test, the null hypothesis (i.e., no difference in the visual reweighting for the improved participants between EMS and visual groups) was rejected with *p* = 0.014, indicating that the visual reweighting in the EMS group was significantly higher than that in the visual SA group.

**Fig 8:**
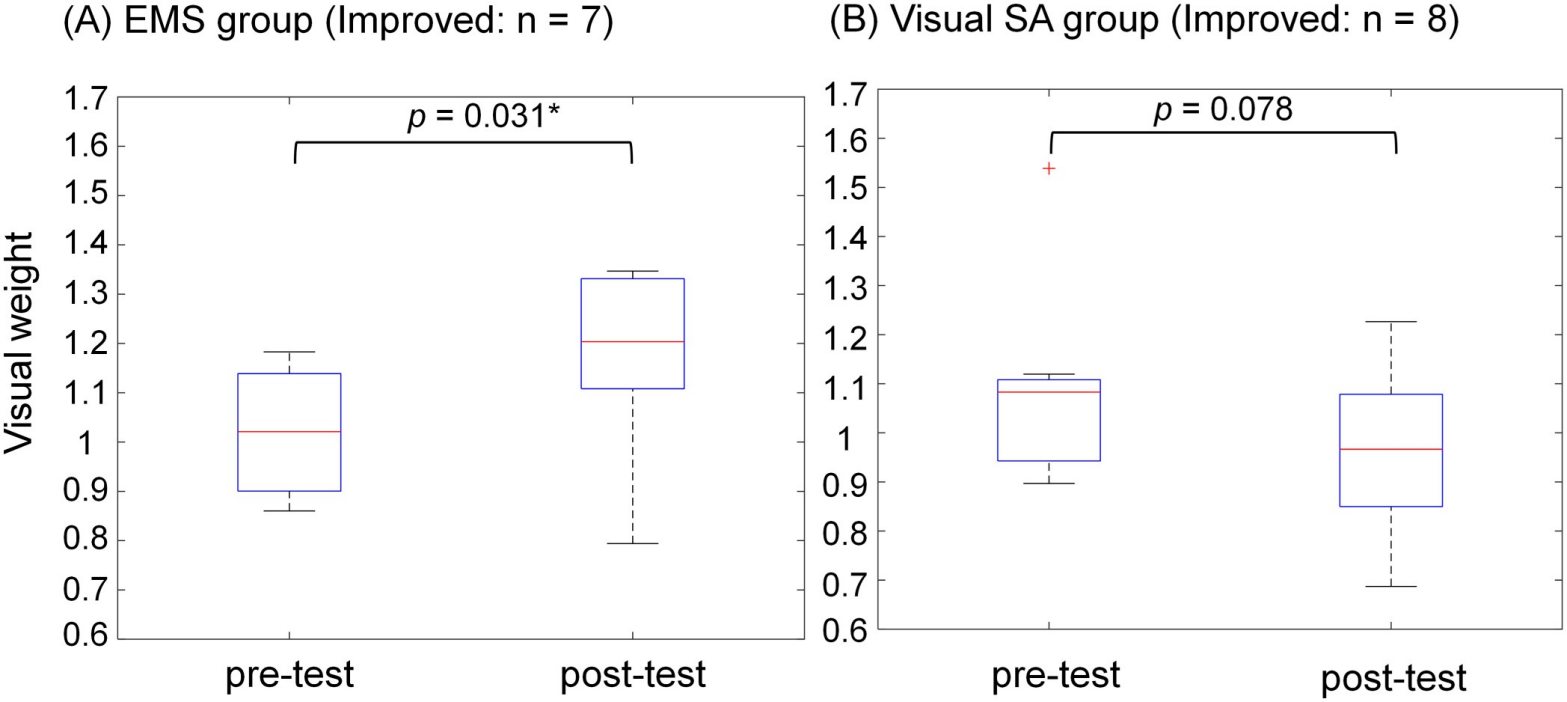
Box plots of visual weight (*W*_*vis*_) between pre- and post-tests in those who improved their posture in the (A) EMS group (n = 7) and (B) visual SA group (n = 8).

Fig 8 shows the changes in the visual weight (*W*_*vis*_) between the pre- and post-tests for improved participants in the (A) EMS group (n = 7) and (B) visual SA group (n = 8). In the EMS group (*p* = 0.031), the visual weight in the post-test was significantly higher than that in the pre-test, but there was no significant difference in the visual SA group (*p* = 0.078).

### Muscle activity changes and duration of stimulation presentation

To examine the induction process in visual reweighting influenced by both the EMS and visual SA methods in more detail, we assessed changes in muscle activity before and after stimulation and the presentation duration of each stimulation.

**Fig 9:**
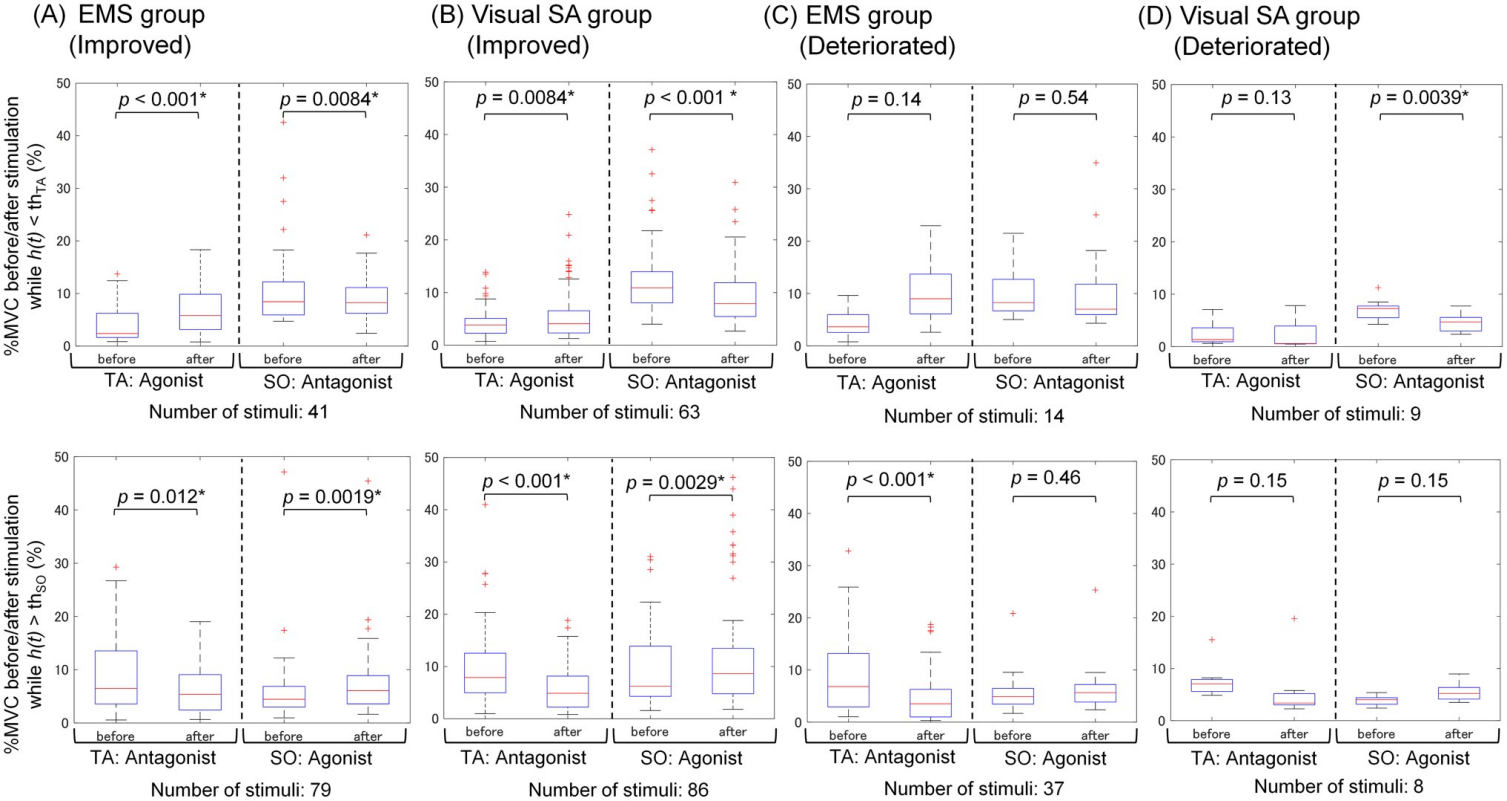
%MVC in the TA and SO before and after stimulation presentation to the TA (upper side) and SO (lower side) for (A) the improved participants in the EMS group, (B) improved participants in the visual SA group, (C) deteriorated participants in the EMS group, and (D) deteriorated participants in the visual SA group. When *h*(*t*) < *th*_*TA*_, participants are required to work with the TA as an agonist muscle and the SO as an antagonist muscle, and when *h*(*t*) > *th*_*SO*_, they are required to work with the TA as an antagonist muscle and the SO as an agonist muscle.

Fig 9 shows the %MVC in the TA and SO before and after stimulation to the TA (upper side) and SO (lower side) for the (A) improved participants in the EMS group, (B) improved participants in the visual SA group, (C) deteriorated participants in the EMS group, and (D) deteriorated participants in the visual SA group. For stimulation when *h*(*t*) < *th*_*TA*_, the participants are required to work with the TA as an agonist muscle and the SO as an antagonist muscle. In contrast, for stimulation when *h*(*t*) > *th*_*SO*_, the participants are required to work with the TA as an antagonist muscle and the SO as an agonist muscle. In the improved participants in both the EMS and visual SA groups, the value of %MVC in the agonist muscles after each stimulation was significantly higher than that before stimulation, and the value of %MVC in the antagonist muscles after each stimulation was significantly lower than that before stimulation. In the deteriorated participants in the EMS group, the only significant difference was in the TA that worked as an antagonist muscle when *h*(*t*) > *th*_*SO*_. In the deteriorated participants in the visual SA group, the only significant difference was in the SO that worked as an antagonist muscle when *h*(*t*) < *th*_*TA*_.

**Fig 10:**
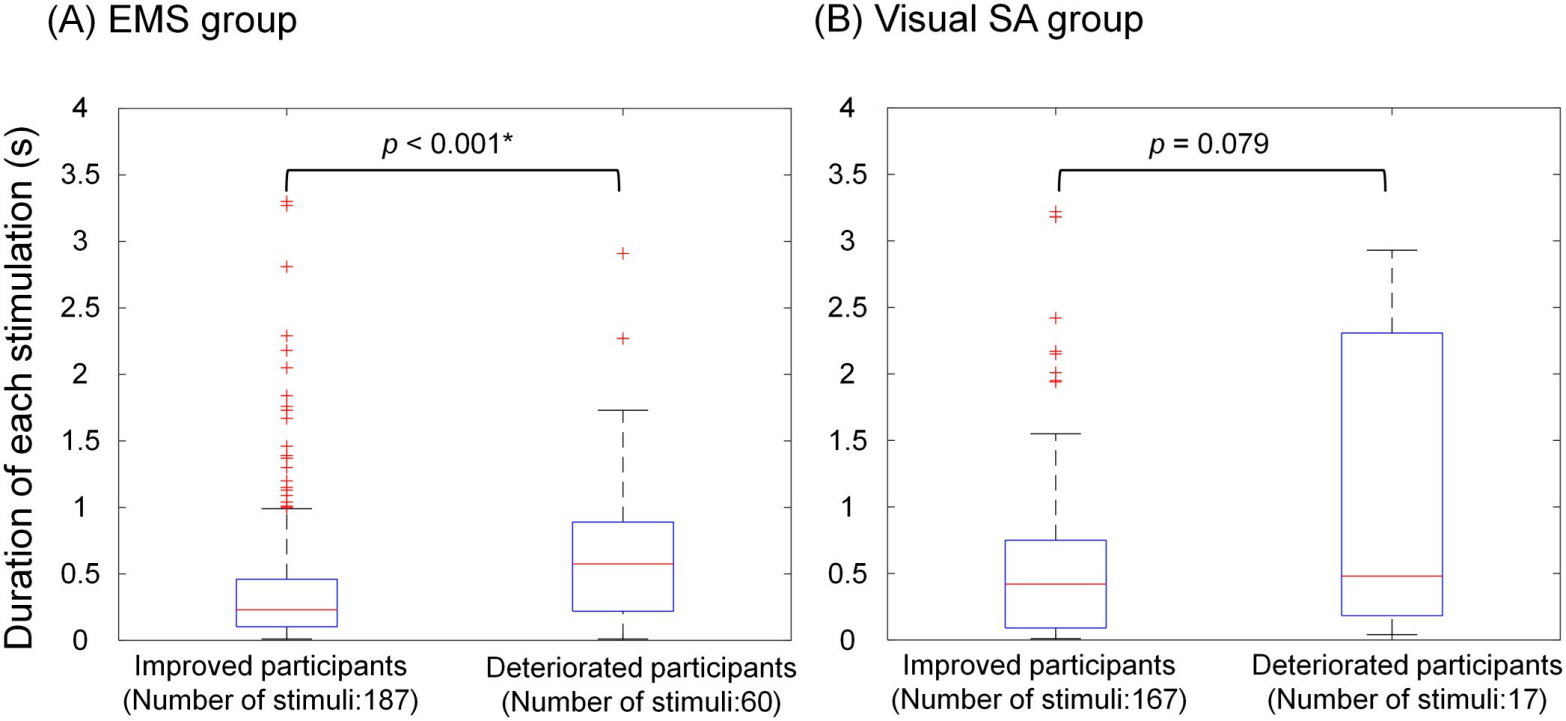
Duration of each stimulation in the stimulation test for improved and deteriorated participants in the (A) EMS group and (B) visual SA group.

Fig 10 shows the duration of each stimulation in the stimulation tests of the improved and deteriorated participants in both the (A) EMS group and (B) visual SA group. The number of stimuli indicates the sum of those for each participant. A shorter duration means the participants moved the balance board back to the near-horizontal non-stimulation area, resulting in a longer time in a stable posture. In the EMS group, the duration of each stimulation was significantly shorter in the improved participants than in the deteriorated participants (*p* < 0.001). In the visual SA group, there was no significant difference between the improved and deteriorated participants (*p* = 0.079).

## Discussion

In this study, we investigated the difference in the induced effect of EMS and visual SA on sensory reweighting dynamics while participants were standing on a balance board rotating in the anteroposterior direction. The results of the linear regression between the visual reweighting and balance-board sway ratio (Fig 5) revealed that the slope of the regression line was negative and positive for EMS and visual SA, respectively. This demonstrates that the tendency of sensory reweighting dynamics to reduce the balance-board sway differs depending on the stimulation method. Moreover, for improved participants, the visual reweighting between the EMS and visual SA groups (Fig 7) was significantly different, demonstrating that the induced effect on sensory reweighting dynamics also differs in accordance with the stimulation methods.

### Induction process of sensory reweighting dynamics for improved participants

First, we discuss the induction process of visual up-weighting for improved participants in the EMS group, as demonstrated in the association between visual reweighting and the balance-board sway ratio (Fig 5). The EMS is presented to either ankle dorsiflexion or plantarflexion muscles to involuntarily control the balance-board tilt horizontally using these muscles. As shown in Fig 9(A), the muscle activity (%MVC) of these muscles before and after EMS for the improved participants showed the activation of the agonist muscle and suppression of the antagonist muscle. In the balance-board task, simultaneous antagonistic co-contraction increases the rigidity of the ankle joints due to the spinal reciprocal inhibition failure [30], which may prevent the balance-board tilt from returning horizontally. EMS plays a role in facilitating reciprocal inhibition against muscles opposite to those to which the EMS is presented, as it has been used to reduce spasticity associated with central nervous system disorders (such as stroke and spinal cord injury [31]) and to promote muscle stretching in healthy subjects [32]. In light of this knowledge, our EMS presented to agonist muscles may suppress antagonist muscles, resulting in the stabilization of the balance-board sway. Because this involuntary ankle control by EMS helps participants with voluntary ankle control, EMS can reduce their attention around the ankle to maintain a horizontal balance-board tilt. Therefore, participants with a reduced balance-board sway due to EMS may rely more on visual information other than ankle proprioception to refer to the horizontal plane.

Second, in the visual SA group, the association between visual reweighting and the balance-board sway ratio showed that the participants’ down-weighted vision improved, in contrast to those in the EMS group (Fig 5). Our visual SA provides instruction cues regarding ankle control through visual inputs. As shown in Fig 9(B), the muscle activity in the ankle dorsiflexion or plantarflexion muscle before and after the visual stimulus for improved participants showed activation of the agonist muscle and suppression of the antagonist muscle after stimulus, similar to EMS. However, these changes in muscle activity were accomplished through independent voluntary ankle control by the participants because visual SA does not induce involuntary ankle control. Thus, our visual SA may increase attention to voluntary ankle control to maintain a horizontal balance board. This increased attention to the ankle suggests that the participants up-weighted ankle proprioception and down-weighted vision. However, we need to consider why there was no significant difference in the visual weight between the pre- and post-tests in Fig 8(B). Our visual SA that presents motor commands on a monitor may not have been enough to direct attention to the ankle. Constant monitoring of the time series of the board sway is likely to induce bigger visual weighting changes, since subjects have to pay attention to detailed ankle control even if they are standing within the stability zone.

We presume that this difference in the induced effect on sensory reweighting dynamics between EMS and visual SA is due to the alteration of the degree of attention to ankle control by each stimulation (i.e., EMS reduced attention while visual SA increased it). A previous study on controlling attention to stabilize posture suggested that a different sensory reweighting could be induced depending on the body part [33]. Moreover, a related study on vibrotactile SA around the trunk showed a greater increase in reliance on vestibular inputs [11]. Since the vestibular function controls head and trunk movements [34], the vibrotactile SA contributes to vestibular up-weighting. These findings support our hypothesis that stimulation alters the degree of attention and induces different sensory reweighting tendencies.

### Difference in induced effects on sensory reweighting dynamics between improved and deteriorated participants

First, in the EMS group, the association between visual reweighting and the balance-board sway ratio revealed that the deteriorated participants had a down-weighted vision (Fig 5). As shown in Fig 9(C), the muscle activity before and after EMS showed similar changes in the improved participants, but it was only significant in the case of the suppression of the antagonist muscle (TA) when EMS was applied to the SO. Although the small sample size makes it difficult to speak with certainty, it is clear that the deteriorated participants could not control switching between agonist and antagonist muscles. The longer duration of each stimulus, as shown in Fig 10(A), means the participants took a longer time from the beginning to tilt to the stimulation area and return to the near-horizontal non-stimulation area. In addition, considering the original highest visual weight observed in the deteriorated participants, as shown in Fig 6(A), they may originally had a low proprioceptive weight. Low reliance on proprioceptive inputs results in a smaller response in the foot or ankle-joint area [2]. We assume that these participants were less responsive to information about the ankle movements required to control the balance board tilted horizontally by EMS.

Second, in the visual SA group, the association between visual reweighting and the balance-board sway ratio revealed that the deteriorated participants had up-weighted vision (Fig 5), and they originally had the lowest visual weight (Fig 6(B)). Our visual SA with instructions for ankle-joint movements encourages using the ankle joint. A related study that assessed the relationship between the use of the lower limb and sensory reweighting implied that low visual weight was associated with less use of the ankle joint [35]. Therefore, we assume that the ankle-control instructions from visual SA made the task more challenging for deteriorated participants.

### Limitations

There were a few limitations in this study. First, we did not quantitatively evaluate any proprioceptive and vestibular reweighting dynamics other than vision. For a more in-depth understanding of the induced effects on sensory reweighting, with the ultimate aim of developing a new postural training system, we encourage further investigation into other sensory reweighting dynamics. Second, we did not evaluate a group under the no-stimulation condition because we aimed to evaluate the difference in the reweighting dynamics associated with postural sway reduction through stimulation methods (EMS and visual SA). To determine the validity of the induced effects of the stimulations in more detail, both the comparison of the reweighting dynamics and the effect of postural sway reduction between the no-stimulation group and the stimulation group should be examined. The final limitation concerns the number of participants and gender bias. In the former, we could not statistically evaluate the deteriorated participants due to the small sample size. We will thus increase the number of participants to help clarify the characteristics of these participants in the future. As for the latter, we did not recruit female participants due to the complication of gender differences related to muscle contraction and pain stemming from differences in body composition [36]. To ensure the effectiveness of the stimulation, we limited the subject characteristics at this time. We plan to solve the problem by using large electrodes and changing the stimulation parameters.

## Conclusion

In this study, we investigated the difference in the induced effects of EMS and visual SA on the sensory reweighting dynamics of participants standing on a balance board rotating in the anteroposterior direction. EMS was presented to the ankle dorsiflexion or plantarflexion muscles based on the balance-board tilt, and the visual SA was presented via a front monitor based on the balance-board tilt. Our results indicate that the tendency of sensory reweighting dynamics to reduce the balance-board sway differs depending on the stimulation method used. Moreover, for those whose postural sway was reduced by each stimulation, we confirmed that the induced effect on sensory reweighting dynamics differs in accordance with the stimulation method used (visual up-weighting in the EMS group and down-weighting in the visual SA group). These different induced effects may be due to the alteration in the degree of attention to ankle control by each stimulation. Our findings suggest there is an appropriate stimulation method to induce the targeted sensory weights; that is, postural training using EMS may be effective for users who have excessively low visual weight or excessively high other sensory weight, while postural training using visual SA may be effective for users who have excessively high visual weight or excessively low other sensory weight. Future investigation into the relationship between sensory reweighting dynamics and stimulation methods will contribute to the proposal and implementation of a new training method that enables users to select the appropriate stimulation method to control the target sensory weights, which can be applied to a wide range of areas including fall prevention, adaptation to physical disorders, sports training, and motion-sickness prevention.

## Supporting information

S1 Data File of the minimal data set used in the experimental results. (XLSX)
